# Branching process approach for epidemics in dynamic partnership network

**DOI:** 10.1007/s00285-017-1147-0

**Published:** 2017-06-01

**Authors:** Abid Ali Lashari, Pieter Trapman

**Affiliations:** Department of Mathematics, Stockholm University, 106 91 Stockholm, Sweden

**Keywords:** *SI* epidemic, Branching process, Basic reproduction number, Dynamic network, Stochastic epidemic model, 60J80, 92D30

## Abstract

We study the spread of sexually transmitted infections (STIs) and other infectious diseases on a dynamic network by using a branching process approach. The nodes in the network represent the sexually active individuals, while connections represent sexual partnerships. This network is dynamic as partnerships are formed and broken over time and individuals enter and leave the sexually active population due to demography. We assume that individuals enter the sexually active network with a random number of partners, chosen according to a suitable distribution and that the maximal number of partners that an individual can have at a time is finite. We discuss two different branching process approximations for the initial stages of an outbreak of the STI. In the first approximation we ignore some dependencies between infected individuals. We compute the offspring mean of this approximating branching process and discuss its relation to the basic reproduction number $R_0$. The second branching process approximation is asymptotically exact, but only defined if individuals can have at most one partner at a time. For this model we compute the probability of a minor outbreak of the epidemic starting with one or few initial cases. We illustrate complications caused by dependencies in the epidemic model by showing that if individuals have at most one partner at a time, the probabilities of extinction of the two approximating branching processes are different. This implies that ignoring dependencies in the epidemic model leads to a wrong prediction of the probability of a large outbreak. Finally, we analyse the first branching process approximation if the number of partners an individual can have at a given time is unbounded. In this model we show that the branching process approximation is asymptomatically exact as the population size goes to infinity.

## Introduction

Sexually transmitted infections (STIs) are among the world’s most common diseases remaining as a major global threat. In addition to accounting for millions of deaths so far, over a million STIs are acquired every day worldwide, and STI pandemics continues to cause a major socio-economic burden on many developing countries (see, e.g. WHO 2015).

Over the past decades, several authors have used mathematical models to asses the impact of the structure of partnerships on the spread of HIV (Eaton et al. 2011; Heesterbeek et al. 2015). See also the Introduction of the Ph.D. thesis of Leung (2016) for an excellent discussion. In order to study the disease dynamics of HIV and other infectious diseases, much attention has been devoted to static networks (see e.g. Newman 2002; Diekmann et al. 2013; Ball et al. 2010, and references therein). The underlying assumption of that work is that once a connection is formed between two individuals this will remain unaltered and during an epidemic outbreak no new partnerships are formed. However, social interactions do often vary over time: new connections being formed and others being dissolved, providing short term opportunities for disease transmission. To incorporate the dynamics, Leung et al. (2012) (see also Leung 2016; Leung and Diekmann 2017) developed and analysed a deterministic model for the spread of an *SI* epidemic on a dynamic network. Here *S* stands for susceptible and *I* stands for infective. Their network model incorporates demographic turnover through individuals entering the population and dying and allows for individuals to have multiple partners at the same time, with the number of partners varying over time. This network model can be seen as an extension of pair formation models (Kretzschmar and Dietz 1998) to situations where individuals are allowed more than one partner at a time. Leung et al. (2012, 2015) extended the traditional pair formation models by incorporating the assumption that individuals have at most *n* partners at a given time.

A key parameter in epidemic modelling is the basic reproduction number, $R_0.$ In epidemics on networks, it is usually defined as the expected number of secondary infections caused by a typical case in the early stages of the epidemic, but not the initial case, in a predominantly susceptible population. This concept is used both in deterministic and stochastic models for infection spread (Diekmann et al. 2013). It is well known that for a susceptible-infectious-recovered (SIR) epidemic in a homogeneously mixing population the process describing the number of infectious individuals during the early stages of the epidemic is well approximated by a suitable branching process (Ball and Donnelly 1995). In those branching process approximations, giving birth corresponds to infecting someone and death corresponds to actual death or recovery, while $R_0$ corresponds to the offspring mean in the branching process. In particular, if $R_0 \le 1$, then no epidemic is possible, while if $R_0>1$ the probability of a large outbreak is strictly larger than 0, but often strictly less than 1. There has been a lot of research on analysing the epidemic threshold, i.e. $R_0=1$, by rigorous branching approximation for the stochastic epidemic models involving networks (see, e.g. Britton 2010, and references therein). In fact, the technique of Ball and Donnelly (1995) can be used to approximate the initial phase of an epidemic on the contact network that has a large size by a suitable branching process (see, e.g. Ball et al. 2009, 2014).

The present study is an extension of the work of Leung et al. (2015) and Leung (2016). Leung and co-authors use deterministic models to study different epidemic models on the dynamic graphs introduced in their work (that we briefly discuss in the following paragraph). In this deterministic approach, one implicit assumption is that the initial fraction of the population which is infectious might be very small, but always positive, which implies that the number of initially infectious individuals is large, because it is effectively assumed that the total population size is infinite. In the present study, we consider the epidemic and population dynamics as stochastic processes, where the expected population size is large but finite.

The network model of Leung et al. (2015) and Leung (2016) can be described as follows (for a detailed description see Sect. 2). Individuals enter the population at rate $\mu N$ and die at rate $\mu$ per individual. This implies that the population size converges to *N*, which is assumed to be very large (and in the deterministic models effectively chosen to be infinite). Individuals enter the population without partners. An individual has at most *n* partners at a time, where *n* is a strictly positive integer (and can be chosen to be $\infty$). The possible partnerships are represented by so-called binding sites. At time *t*, let $(1-F(t))n$ be the average number of partners per individual in the population, i.e. *F*(*t*) is the fraction of binding sites that are “free” at time *t*. If an individual has *k* partners at time *t* it acquires a new partner at rate $(n-k)\rho F(t),$ where $\rho$ is a constant (the rate at which each free binding site tries to connect with another binding site; and that site is chosen uniformly from all binding sites and is thus free with probability *F*(*t*)) and partners separate at rate $\sigma$ per partnership. In the *SI* epidemic framework, a susceptible individual becomes infectious at a rate $\beta$ times the number of his or her infectious partners. Infectious individuals cannot recover, but of course they stop spreading when they die. A key ingredient in the models of Leung et al. (2015) is the mean-field at distance one assumption, which is a (non-exact) approximation of the distribution of the number of partners of partners of a newly-infected individual.

We approach the models by Leung and co-authors from a stochastic perspective. To do this, we make some further assumptions, which make computations easier and the communication of our main message clearer. In contrast to the deterministic models mentioned before, we do not assume that a new individual in the population starts as single. Instead, we assume that the individuals upon entering the population immediately form a (random) number of partnerships with individuals already in the population. The distribution of this random number is chosen in such a way that the *distribution* of the number of partners of an individual does not change over time. That is to say, incoming individuals have a stationary distribution of the number of partners (usually referred to as the degree distribution). The advantage of this assumption is that dependencies between the number of partners of an individual and the infection status of the individual become more tractable and the mean-field at distance one approximation of Leung et al. (2015) and Leung (2016) is no longer needed. We follow Leung et al. (2015) to ignore the difference between male and female in our model and in this way effectively consider a homosexual or asexual population. Although this might be unrealistic, we think our main message is highlighted clearer by this omission.

The main purpose of this paper is to analyse possible approximations of the early stages of a stochastic epidemic in the described network by suitable branching processes. Our analysis focuses on the early stage of an epidemic outbreak where only a small number of individuals is initially infected. Note that this assumption does not fit within the deterministic framework, where the number of initial infectives is either exactly 0 or large, because in those models the initial fraction of the (effectively infinite) population infected has to be either 0 or strictly positive. In particular, we are concerned with deriving explicit formulas for the threshold parameter $R_0$ and the probability of extinction. For this, we use two approximations for the model.

In the first approximation we consider a general maximal number of partners, *n*, but because of certain dependencies to be described in detail afterwards, it is not possible to do more than computing $R_0$, which is here purely interpreted as the expected number of other individuals infected by one infectious individual during the early stages of the epidemic. We note that we are not able to prove that this $R_0$ has the desired threshold property which it has for epidemics in homogeneously mixing populations.

The second approximation is only valid for $n=1,$ which corresponds to the pair formation model of Kretzschmar and Dietz (1998). What makes this approach different from the first is that here we can describe the dynamics of the disease through an asymptotically exact approximating branching process. From this we can easily obtain the extinction probability as well as a threshold parameter, denoted by $\hat{R}_0.$ This reproduction number $\hat{R}_0$ differs from $R_0$ and cannot be interpreted as the expected number of individuals infected by a typical infected individual. The interpretation of $\hat{R}_0$ is discussed in Sect. 3.2. Unfortunately, we did not find a way to generalize this approach to $n>1$. For further reflections on $R_0,$ we refer the reader to Cushing and Diekmann (2016).

Finally, in order to avoid undesirable dependencies that appear and complicate the two branching process approximations, we also study the case in which there is no maximal number of partners, i.e. when $n = \infty$ (cf. Altmann 1995). For this model, we can compute the reproduction number as well as an implicit expression for the extinction probability.

The main contributions of the current work are:

- to present a branching process approach for analysing the early stages of an outbreak of a sexually transmitted infection, or any other infectious disease, spreading along the dynamic network. In doing this, we show why an appealing straightforward branching process approximation of the epidemic process is not correct, because it ignores some subtle dependencies.
- to characterize the basic reproduction number and the probability of extinction for the dynamic network by using a branching process approach.

The paper is structured as follows. Section 2 is devoted to the model definition and assumptions. In Sect. 3, we present two stochastic approximations of the model. In the first, we use a naive (appealing but wrong) branching process approximation to analyse the early phase of an epidemic spreading through a dynamic sexual network. We use the second (less intuitive) approximation of the model with $n=1$ to compute a threshold parameter $\hat{R}_0$ and the correct probability of extinction during the initial phase of the epidemic. Here we also provide a discussion of the influence of the dependencies. In Sect. 4, the first approximation of the model is used to study the epidemic on the dynamic network when the partnership capacity is infinite, i.e. when $n= \infty$. In this particular case, dependencies fall away and we may use branching processes to analyse the early phase of an *SI* epidemic spreading through a dynamic sexual network. In particular, we compute the reproduction number $\mathbb {R}_0,$ the offspring distribution and compare reproduction numbers when $n\rightarrow \infty .$ Finally, we discuss our analytical findings and give an outlook on future work in Sect. 5.

## Model definition and assumptions

In our model we assume that individuals enter the population at rate $\mu N$ (i.e. according to a Poisson process with intensity $\mu N$) and that individuals have independent exponentially distributed “lifetimes” (or time they stay in the active population), with expectation $1/\mu$, i.e. individuals leave the active population at rate $\mu$ times the number of individuals in this population. This implies that the distribution of the population size, say $N^*(t)$, converges as $t\rightarrow \infty$ to a Poisson distribution with mean *N*, i.e. the stationary and limiting distribution of the population size is Poisson distributed with expectation *N* (Resnick 2013, Ch. 5). We assume that *N* is very large.

When an individual enters the population, he or she immediately forms partnerships with a random number of partners. This random number of partners is independent for different individuals and binomially distributed with parameters *n* and $p_{\text {in}}$, where *n* is a positive integer, representing the maximal number of partners an individual can have at any given time (the partnership capacity) and $p_{\text {in}}$ is a constant between 0 and 1, to be specified later. So, the probability that an entering individual has $\ell$ partners is $$\left( {\begin{array}{c}n\\ \ell \end{array}}\right) (p_{\text {in}})^{\ell }(1-p_{\text {in}})^{n-\ell }$$. The probability that the incoming individual forms a partnership with an individual that already has *k* partners at that moment is proportional to $n-k$. A given individual with $\ell$ partners acquires new partners among the individuals already in the population at rate $(n-\ell ) \rho F(t)$, where *F*(*t*) is the fraction of binding sites free at time *t*. Again, the probability that a partnership is formed with an individual that at that moment already has *k* partners is proportional to $n-k$. Note that we can interpret this construction as follows: a given individual with $\ell$ partners and a given individual with *k* partners form a partnership at rate $(n-\ell )(n-k)\rho /(n N)$. Partnerships have independent exponential durations with expectation $1/\sigma$, i.e. partners separate at rate $\sigma$ per partnership (if the partnership has not ended by death of one of the partners). If an individual leaves the active population, then all of its partnerships break.

From a modelling perspective individuals can be seen as collections of *n* “binding sites”, where binding sites can either be free or occupied (by a partner). As long as individuals are alive, their binding sites behave independently where partnership formation and separation is concerned. Let *F*(*t*) be the fraction of binding sites in the population which are free at time *t*. We want this fraction to converge (with high probability) to a constant *F*,  which we use in the formulation of the branching process. Observe that, because the number of partnerships of an individual just after entering the population is binomially distributed with parameters *n* and $p_{\text {in}},$ as a result, we can consider the binding sites of such an individual to be independent and free with probability $1-p_{\text {in}}$. We choose $p_{\text {in}}$ such that *F* is equal to $1-p_{\text {in}}$, because that is a necessary condition for the distribution of the number of partners of an individual to be stationary. Note that if a binding site is occupied it becomes empty at rate $\sigma + \mu$, where the $\sigma$ term is caused by separation and the $\mu$ term is caused by death of the partner. A binding site, that is already in the population, acquires new partners already present in the population at rate $\rho F(t).$ The rate at which occupied binding sites enters the population is $\mu Nn p_{\text {in}}$. The number of free binding sites in the population is $F(t) N^*(t) n.$ Therefore, per binding site, the rate of acquiring newly arrived partners is $\frac{\mu Nn p_{\text {in}}}{F(t)N^*(t)n}.$ So an empty binding site acquires a new partner at rate $\rho F(t) + \frac{\mu N n p_{\text {in}}}{n N^*(t) F(t)}$. If *F*(*t*) indeed converges to $F=1-p_{\text {in}}$ (and using the fact that $N^*(t)/N$ converges in probability to 1 as $N\rightarrow \infty$), then the rate of acquiring a new partner at a binding site is well approximated by $\rho F + \frac{\mu (1-F)}{F}$.

Putting the above together with the theory of Markov on–off processes (Resnick 2013, p. 405), the long run fraction of binding sites which are free is given by

$$\begin{aligned} \frac{\sigma + \mu }{\sigma + \mu + \rho F + \frac{\mu (1-F)}{F}} = \frac{(\sigma + \mu ) F}{\mu + \sigma F + \rho F^2}. \end{aligned}$$

This fraction should be equal to *F*. As a result,

1 $$\begin{aligned} \rho F^2 = \sigma (1-F) \end{aligned}$$

or

2 $$\begin{aligned} F=\frac{-\sigma +\sqrt{\sigma ^{2}+4\rho \sigma }}{2\rho }. \end{aligned}$$

(Clearly the other solution of (1) is negative.)

So, we choose $p_{\text {in}} = 1-\frac{-\sigma +\sqrt{\sigma ^{2}+4\rho \sigma }}{2\rho }$. The parameters of our model are summarized in Table 1.

**Table 1:** The descriptions of the parameters for the model (1)

*N*	Expected number of individuals in population
*n*	Number of binding sites per individual
$\rho /(nN)$	Rate of making attempts of new connections per pair of free binding sites
$\mu$	Natural mortality rate per individual
$\sigma$	Separation rate per partnership
$\beta$	Disease transmission rate per partnership
*F*(*t*)	Fraction of free binding sites at time *t*
$p_{\text {in}}$	Fraction of binding sites of new individuals which are occupied

Because the probability that a binding site is free is *F*(*t*) and whether or not a binding site is free is independent of other binding sites, the number of partners of a living individual is binomially distributed with parameters *n* and $1-F(t)$, i.e. the number of partners of a living individual is *k* with probability $$\left( {\begin{array}{c}n\\ k\end{array}}\right) (1-F(t))^{k}(F(t))^{n-k}$$ (for $k=0,1,\ldots ,n$). Furthermore, for a given individual assuming that the individual does not die, the transitions of the number of partners are described by

$$\begin{aligned} \begin{aligned} k&\longrightarrow k+1 ~\text {with rate}~ \left( \rho F(t)+\frac{\mu (1-F(t))}{F(t)}\right) (n-k),\\ k&\longrightarrow k-1 ~\text {with rate}~ (\sigma +\mu )k. \end{aligned} \end{aligned}$$

In the following lemma (the proof is presented in “Appendix A”, we show that *F*(*t*) indeed converges (in a suitable sense) to *F* as time *t* and the population size parameter *N* tends to infinity.

### Lemma 1

As $N \rightarrow \infty$ the fraction of free binding sites *F*(*t*) satisfies (on every bounded interval with probability tending to 1) the differential equation

3 $$\begin{aligned} \begin{aligned} \frac{dF(t)}{dt} = -\rho F^{2}(t)+\sigma \left( 1-F(t)\right) -2 \mu \left( p_{\text {in}}- (1-F(t)\right) . \end{aligned} \end{aligned}$$

It is not hard to see that the asymptotically stable equilibrium solution of the differential equation (3) is

$$\begin{aligned} F(t) = \frac{-(\sigma +2\mu )+\sqrt{(\sigma +2\mu )^{2}+4\rho (\sigma +2\mu \left( 1-p_{\text {in}})\right) }}{2\rho }. \end{aligned}$$

So, by filling in $1-p_{\text {in}} =F = \frac{-\sigma +\sqrt{\sigma ^{2}+4\rho \sigma }}{2\rho }$ [see Eq. (2)] and after some trivial computation we have that, for $t\rightarrow \infty$

$$\begin{aligned} F(t)\rightarrow \frac{-(\sigma +2\mu )+\sqrt{(2 \mu + \sqrt{\sigma ^{2}+4\rho \sigma })^2}}{2\rho } = \frac{-\sigma +\sqrt{\sigma ^{2}+4\rho \sigma }}{2\rho } = F. \end{aligned}$$

In the following analysis we assume that the population has already reached equilibrium and *F*(*t*) can be replaced by the constant *F*.

Next, we consider an *SI* epidemic spreading on the dynamic network described above. In this *SI* model, pairs of individuals make contacts according to independent Poisson processes with per partnership intensity $\beta$, as long as the pair is in a partnership. If a susceptible individual contacts an infectious one, it becomes infectious immediately and stays so until it leaves the population. We assume that the infection is introduced in the population by a single infectious individual, when the distribution of the configuration of the network is stationary. All other individuals are at that moment susceptible. With some abuse of terminology, we say that a binding site is susceptible (respectively infectious) if the partner it is connected to (if any) is susceptible (respectively infectious).

In the next section we approximate the spread of an *SI* epidemic on the dynamic network by a branching process. For this approximation we need some further assumptions and notations. In this branching process approach, we keep track of properties of the infectious individuals and their binding sites. We implicitly assume that the number of susceptible individuals that are not connected to infectious individuals is very large and their properties, such as the distribution of the number of other susceptible partners etc., does not change as long as the branching process approximation is valid, i.e. we study the initial phase of the epidemic.

**Fig. 1:**
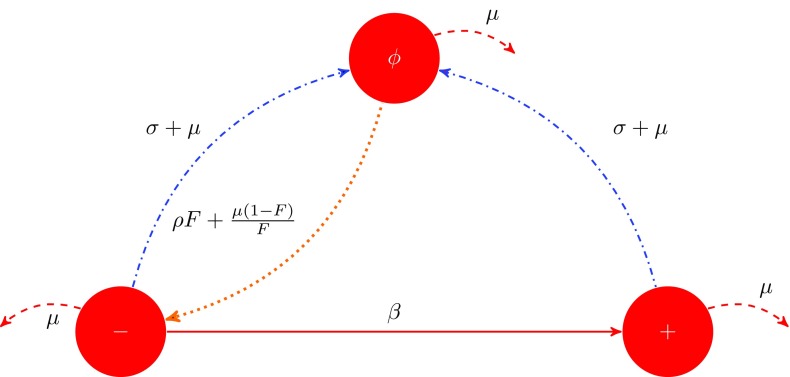
Flow chart describing the possible transitions of a binding site between states $(\phi )$, $(+)$, $(-)$ and their corresponding rates. The continuous *red line* represents transmission of the infection while the *dashed lines* represents death. The *dash-dotted blue lines* represents occupied binding site becoming free while the *dotted orange line* represents free binding site becoming occupied

The possible states of a binding site of an infectious individual are: free (denoted by $\phi$) or occupied by a susceptible (denoted by −) or occupied by an infectious individual (denoted by $+$). The binding sites of an individual move among the possible states according to a Markov process. The disease is transmitted from an infectious partner to a susceptible partner at rate $\beta$. Such a transmission causes a transition of the state of the binding site from − to $+$. Other possible transitions are from − or $+$ to $\phi$, which both happen at rate $\sigma + \mu$ (end of partnership or death of partner) and from $\phi$ to − at rate $\rho F+\frac{\mu (1-F)}{F}$ (formation of new partnership; the new partner being susceptible with high probability). Finally, the dynamics of this particular Markov process stops by death of the infectious individual under consideration, which happens at rate $\mu$. The states and the transitions of this Markov process are shown schematically in Fig. 1.

## Branching process approaches to the spread of epidemics

### A first naive approach

In this subsection, we study the spread of an STI (or other infectious disease) on the partnership network in the beginning of an epidemic by employing an appealing but wrong branching process approximation approach. In the approximating branching process dependencies, which are present in the spread of an epidemic on a network, are ignored. However, we still present this approximation because the approximation is so appealing. Furthermore, the dependencies that are ignored in the branching process approximation are also ignored in deterministic descriptions of the epidemic (see Leung et al. 2015; Leung and Diekmann 2017). We highlight dependencies that are present and how ignoring those dependencies leads to wrong predictions of the probability of a large outbreak.

Here, we assume that everyone has *n* binding sites (i.e. an individual has at most *n* partners at the same time). In the present approach, the dynamic network model can be seen as a discrete space, continuous time Markov chain. So, we can describe the dynamics of the process in terms of rates (depending on the current state of the population), where times between events are exponentially distributed.

Recall that an infectious individual can produce new infectious binding sites through contacts at his or her susceptible binding sites. The number of new infections caused by one infectious individual is the same as the sum of the number of times we have a transition from the − state to the $+$ state, where the sum is taken over all binding sites of the infectious individual. Thus, in our consideration a child is born (i.e. an infectious binding site is created) whenever there is a passage from the–state to the $+$ state. In the terminology of Galton–Watson branching processes (Jagers 1975), infectious binding sites generated by an infectious individual are considered as his or her offspring. However, we stress again, as we show later, that the epidemic process is not approximated by this branching process in the sense that it is not asymptomatically exact.

In “Appendix B” we derive an offspring distribution for the branching process, which then leads to an expression for the offspring mean and the probability of extinction of the branching process. Which, if the branching process approximation is asymptotically exact, correspond respectively to the basic reproduction number $R_0$ and the probability of a minor outbreak. Let $R_0$ be the offspring mean of the branching process, we deduce that

4 $$\begin{aligned} R_0 =\displaystyle \frac{\beta (1-F)}{\mu (\sigma +\mu +\mu F)(\beta +\sigma +2\mu )}\left( (\sigma +\mu )^{2}+ (n-1)(\sigma +2\mu )(\sigma +\mu +\mu F)\right) . \end{aligned}$$

If $n =1$, the probability of extinction of the branching process can be easily deduced as well (see “Appendix B”). Indeed, the probability of extinction of the branching process, when $n=1$ and the ancestor is one infectious individual without a partner, is given by

5 $$\begin{aligned} q_{\phi } = \frac{1}{R_0}-\frac{\mu (R_0-1)}{(\sigma +\mu )R_0}. \end{aligned}$$

Note that in computing $R_0$ we do not need independence of the number of children at different binding sites (which indeed are not independent). Therefore, this offspring mean is a good approximation for the expected number of new infections caused by an infected individual during the early stages of the epidemic, in a mostly susceptible population. However, we do not know whether there is a branching process approximation of the spread of the epidemic which is asymptotically exact and has the same offspring mean. So we do not know whether $R_0=1$ is a threshold for a large outbreak of an epidemic which starts with only a few infectious individuals.

The number of partners in our model can have a great effect on $R_0$. To see this effect, we assume that the average number of partners of an individual is a constant *C* i.e. $n(1-F)=C$. Using this in (4) and treating *n* as a positive continuous variable, straightforward computation gives

$$\begin{aligned} \frac{\partial R_0}{\partial n} =\frac{\beta C(\sigma +2\mu )(n-C)\left( 2(\sigma +\mu )n+\mu (n-C)\right) }{n^2\left( (\sigma +\mu )n+\mu (n-C)\right) ^2(\beta +\sigma +2\mu )}. \end{aligned}$$

Since *C* is always less than *n*,  the derivative $\frac{\partial R_0}{\partial n}>0,$ i.e. the basic reproduction number $R_0$ increases as a function of the number of partners.

The independence of the number of children of individuals can be viewed as the very defining property of branching processes and we have already emphasized that although the stochastic process leading to (4) and (5) is a branching process, it does not approximate the epidemic process well since the epidemic process violates the required independence criterion of reproducing individuals, even for the simplest case $n=1$. Indeed, information about the state of the partners of one of the individuals provides some information about the state of the partner of other individuals. To understand this, consider what happens if an individual in state $+$ dies. We know with certainty that his or her partner gets a free binding site. While, if two infected partners separate, then we know for sure that both the infected individuals that were in the partnership, get a free binding site at the same time. We further clarify the dependencies that violates the independence criterion of reproducing individuals for $n=1$ through the following example. In this example we use the following probabilities.

$$\pi _{\phi }$$:
Probability that a $\phi$ binding site becomes $+$ before it disappears, i.e. before the individual under consideration dies.
$$\pi _-$$:
Probability that a − binding site becomes $+$ before it disappears.
$$\pi _+$$:
Probability that a $+$ binding site becomes $+$ again after having been − or $\phi$ before it disappears.

It is straigtforward to deduce (see “Appendix B”) that

6 $$\begin{aligned} \pi _+ =\frac{\sigma +\mu }{\sigma +2\mu }\pi _\phi . \end{aligned}$$

#### Example 1

Consider the case when an infector has exactly 1 “child”, the infectee. We consider what happens from the moment of the first infection on.

$$\begin{aligned}&\mathbb {P}\,\text {(infectee has } 0 \text { children }| \text { infector has 1 child)} \\&\quad =(1-\pi _{\phi })\mathbb {P}\,\left( \text {first event after infection is separation }| \text { infector has 1 child}\right) \\&\qquad +\, (1-\pi _{\phi })\mathbb {P}\,\left( \text {first event after infection is death of infector }| \text { infector has 1 child}\right) \\&\qquad +\,\mathbb {P}\,\left( \text {first event after infection is death of infectee }| \text { infector has 1 child}\right) \\&\quad = \frac{\sigma }{\sigma +2\mu } \frac{(1-\pi _{\phi })^2}{1-\pi _{+}} + 2 \frac{\mu }{\sigma +2\mu } \frac{1-\pi _{\phi }}{1-\pi _{+}} = \frac{1- 2 \frac{\sigma + \mu }{\sigma +2\mu } \pi _{\phi } + \frac{\sigma }{\sigma + 2\mu } \pi ^2_{\phi }}{1-\pi _{+}}\\&\quad = \frac{1- 2 \pi _{+} + \frac{\sigma (\sigma + 2\mu )}{(\sigma + \mu )^{2}} \pi ^2_{+}}{1-\pi _{+}} \ne 1-\pi _{+} = \mathbb {P}\,(\text {infectee has 0 children}). \end{aligned}$$

This example shows that there is dependence between the states of the two events even for the simplest case $n=1$.

When $n >1$, the dependencies become even clearer, since when an individual dies all of its partners obtain a free binding site at the same time. Furthermore, whether or not a partner of a partner of an infectious individual is infectious is dependent on how long the individual under consideration has been infectious itself, which creates dependencies between individuals which are not even partners of each other. This observation also means that considering the spread of the epidemic at the level of binding sites (see e.g. Leung and Diekmann 2017) or typing individuals by their own infection status and the number of susceptible and infectious partners they have (cf. Ball and House 2017) is not enough to obtain an asymptotically exact (multi-type) branching process approximation.

### Asymptomatically exact branching process approximation

As stated earlier, we cannot expect that the branching process defined above approximates the epidemic well. Still, this branching process is used to compute the extinction probability in Eq. (5). Therefore, this probability is not necessarily the extinction probability of the epidemic process which is approximated by the branching process. That motivates us for defining a branching process which correctly approximates the epidemic process, so that we can get the true extinction probability for the model when $n=1$. Unfortunately, we do not know how to extend this approach to $n > 1$.

For this branching process, we base our bookkeeping on the empty binding sites. Assume for the moment that we start the epidemic with one infectious individual with binding site in state $\phi$. Now the individual can either die (in which case no new empty binding sites are created), which occurs at rate $\mu$ or form a partnership with a susceptible individual (recall that we are in the early stages of an epidemic), which occur at rate

$$\begin{aligned} \rho F + \mu \frac{1-F}{F} = (\sigma +\mu )\frac{1-F}{F}. \end{aligned}$$

In case of a partnership between an infectious individual and a susceptible individual four things can happen: (i) a separation, in which case there is one infectious individual with an empty binding site which occurs at rate $\sigma$, (ii) the susceptible individual dies, in which case there is also one infectious individual with an empty binding site; this occurs at rate $\mu$, (iii) the infectious individual dies, in which case there is no infectious individual with an empty binding site; this occurs at rate $\mu$ or (iv) the infectious individual infects the susceptible one (rate $\beta$), in which case there is a partnership between two infectious individuals. In creating the branching process approximation below, we consider the resulting infectious individual with an empty binding site in case (i) and case (ii) as new individuals.

**Fig. 2:**
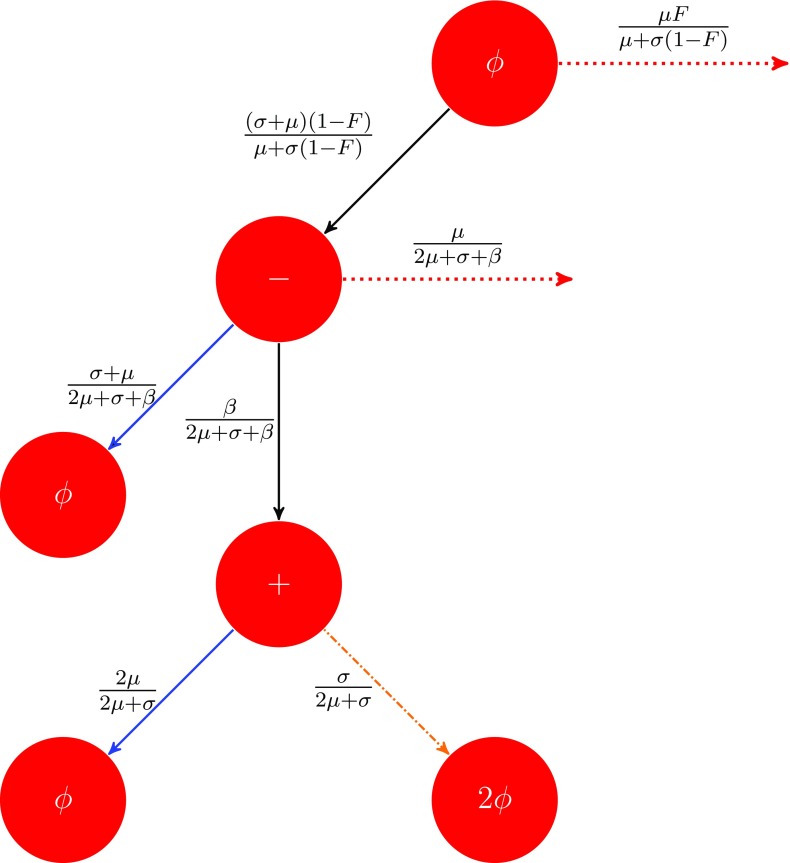
Flow chart describing the offspring of binding site ($\phi$). The *solid black lines* represent “intermediate” transitions of the binding site under consideration, after which the number of new free binding sites produced still depends on further transitions. The *dotted red lines* represents producing 0 offspring, *solid blue lines* represent producing 1 offspring while the *dash-dotted orange line* represents producing 2 offspring. The edge labels are the transition probabilities

In case of a partnership between two infectious individuals two things can happen: (i) a separation, in which case there are two infectious individuals each with an empty binding site, which occurs at rate $\sigma$, (ii) one of the individual dies (rate $2 \mu$), in which case there is one infectious individual with an empty binding site. Again, in creating the branching process approximation below, we consider the resulting infectious individuals with an empty binding site as new individuals. The possible transitions and their rate are schematically depicted in Fig. 2.

Observe that an empty binding site can generate, after possibly going through some stages in which the binding site was occupied, zero, one or two “new” empty binding sites. Here one of the “new” binding sites might actually be the old binding site, which for modelling purposes is considered to be new. To clarify the idea behind our branching process approximation, consider as an example a separation of two infectious individuals. This can be seen as the death of an originally free binding site which paired with and then infected a free susceptible binding site leading to birth of two free binding sites. The “newborn” free binding sites are independent copies of the initial free binding site, which is why this description leads to a branching process approximation of the epidemic spread which is asymptotically exact.

So, each free binding site generates a random number *Y*, $Y\in \{0,1,2\}$, of free binding sites in the next generation, independently of other free binding sites. The probabilities of having *Y* children in the approximating branching process are given by

7 $$\begin{aligned} \begin{array}{rcl}\mathbb {P}(Y=0)&{}=&{}\displaystyle \frac{\mu \left( \sigma +\mu +(\beta +\mu )F\right) }{(\sigma (1-F)+\mu )(\beta +\sigma +2\mu )},\\ \\ \mathbb {P}(Y=1)&{}=&{}\displaystyle \frac{(\sigma +\mu )\left( (\sigma +\mu )(\sigma +2\mu )+2\mu \beta \right) (1-F)}{\left( \sigma (1-F) +\mu \right) (\sigma +2\mu )(\beta +\sigma +2\mu )},\\ \\ \mathbb {P}(Y=2)&{}=&{}\displaystyle \frac{\sigma \beta (\sigma +\mu )(1-F)}{\left( \sigma (1- F)+\mu \right) (\sigma +2\mu )(\beta +\sigma +2\mu )}.\\ \\ \end{array} \end{aligned}$$

This simple interpretation for the branching process is no longer valid if the number of binding sites of an individual exceeds 1, because death of an individual may cause several pairs of infectious individuals to break at the same moment and in that way cause dependencies, which violate the defining properties of branching processes.

For the branching process with an offspring distribution given through the random variable *Y*,  we can compute the offspring mean (which corresponds to the expected total number of new free binding sites generated by one free binding site). We denote this offspring mean by $\hat{R}_0,$ which is given by

8 $$\begin{aligned} \begin{array}{rcl} \hat{R}_0= E(Y)=\displaystyle \frac{(\sigma +\mu )^{2}(\sigma +2\mu +2\beta )(1-F)}{\left( \sigma (1-F)+\mu \right) (\sigma +2\mu )(\beta +\sigma +2\mu )}. \end{array} \end{aligned}$$

Note that this $\hat{R}_0$ is not a basic reproduction number in the biological sense of the word, but as written above, it is a threshold parameter.

For the branching process with offspring distribution *Y*,  we can also calculate the probability of extinction, which we denote by $\hat{q}_{\phi }$. This probability is the minimal solution of the following equation (see Jagers 1975)

$$\begin{aligned} \hat{q}_{\phi } = \mathbb {P}(Y=0) + \mathbb {P}(Y=1)\hat{q}_{\phi } + \mathbb {P}(Y=2)\hat{q}_{\phi }^2, \end{aligned}$$

which is given by

$$\begin{aligned} \hat{q}_{\phi } = \displaystyle \min \left( 1,\frac{\mu (\sigma +2\mu )\left( \sigma +\mu +(\beta +\mu )F\right) }{\sigma \beta (\sigma +\mu )(1-F)}\right) . \end{aligned}$$

Using Eq. (4) and noting that for $n=1$, Eq. (4) gives

9 $$\begin{aligned} \beta = \frac{\mu (\sigma +2\mu )(\sigma +\mu +\mu F)R_0}{(\sigma +\mu )^2(1-F)-\mu (\sigma +\mu +\mu F)R_0}, \end{aligned}$$

we obtain

$$\begin{aligned} \hat{q}_{\phi } = \displaystyle \min \left( 1,\frac{1}{R_0}-\frac{\mu (R_0-1)}{\sigma R_0}\right) . \end{aligned}$$

The $R_0$ in this equation is the basic reproduction number obtained through the naive branching process approximation and does not correspond to the offspring mean of the branching process used to derive $\hat{q}_{\phi }$, still it is useful to use this $R_0$ to simplify the expression for $\hat{q}_{\phi }$. By writing $\hat{q}_{\phi }$ as a function of $R_0$ instead of a function of $\beta$, the explicit dependence of $\hat{q}_{\phi }$ on *F* disappears and we have freedom to choose *F*. However, the denominator in (9) has to be positive and therefore we cannot always choose *F* arbitrary close to 1. Note that the probability of extinction for the approximating branching processes is not the same. In fact, if $R_0>1$, then

$$\begin{aligned} q_{\phi }=\hat{q}_{\phi }+\frac{\mu ^{2}(R_0-1)}{\sigma (\sigma +\mu )R_0}. \end{aligned}$$

**Fig. 3:**
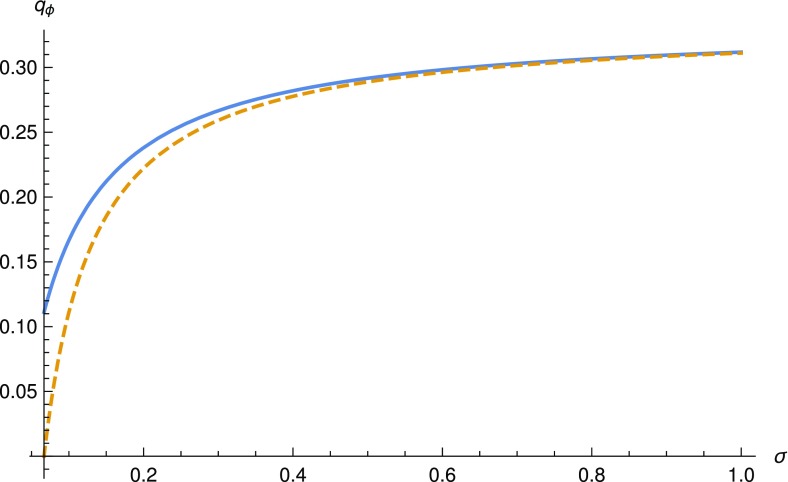
The two extinction probabilities $q_{\phi }$ and $\hat{q}_{\phi }$ for the two branching process approximations of the epidemic process. The *solid line* is obtained using the naive branching process approximation of a minor outbreak $(q_{\phi })$, while the *dashed line* gives the correct probability of a minor outbreak ($\hat{q}_{\phi }$). The *plots* are for $\sigma \ge \mu (R_0-1)$, where $R_0=3$ and $\mu =1/30$

As written earlier, the reason for this is that the first branching process approximation is not a good approximation of the epidemic process because of the dependence between “siblings” and “parents and their children”. In Fig. 3 we compare the two extinction probabilities $q_{\phi }$ and $\hat{q}_{\phi }$ as functions of $\sigma$, where $\sigma \ge \mu (R_0-1)$, while keeping $R_0=3$ and $\mu =1/30$ fixed. We observe that for the given parameter values and for $\sigma$ only slightly above $\mu (R_0-1)$, the difference between the two extinction probabilities is considerable. Note that if $R_0$ is given and when $\sigma$ is only slightly larger than $\mu (R_0-1)$, then *F* is necessarily close to 0, in order for the denominator in (9) to be positive and $\beta$ is necessarily large.

#### Remark

Of particular interest is the critical infection rate $\beta$, denoted by $\beta _c,$ for which $\hat{R}_0=1$, i.e. the minimum of $\beta$ which is necessary to possibly cause an epidemic. We want to know whether $R_0$ is also equal to 1 for this value of $\beta$. It can easily be checked that for $n=1$, indeed both $R_0=1$ and $\hat{R}_0=1$ for

$$\begin{aligned} \beta _c=\displaystyle \frac{\mu (\sigma +2\mu )(\sigma +\mu +\mu F)}{\sigma (\sigma +\mu )(1-F)-\mu F(\sigma +2\mu )}. \end{aligned}$$

This observation, in addition to the deterministic model reproduction number interpretation of $R_0$ given by Leung and co-authors in e.g. Leung et al. (2015), makes us believe that also for $n>1$ a single infected individual can cause a major outbreak with positive probability if and only if $R_0>1$, but we did not find a proof for this.

## Model without maximum partnership capacity

To circumvent the difficulty of dependencies that arises in the branching process approximation for the epidemic process with $n>1$ in the previous sections, we consider the model with $n=\infty$, i.e. the model in which there is no maximal number of partners of an individual. In this model, it is again assumed that new individuals enters the population with a random number of partners where those random numbers are assumed to be independent and identically distributed and chosen in such a way that the number of partners of an individual is stationary during the whole “lifetime”.

In order to avoid a situation where individuals accumulate new partners at infinite speed in $n=\infty$ model, we set for $n<\infty$ the rate at which an individual enters a new partnership per free binding site as $\rho =\frac{\bar{\rho }}{n}$, where $\bar{\rho }$ is a constant. Note that for $n<\infty$, an individual with *k* partners enters a new partnership at rate $(n-k)\rho F$, which would go to infinity if $\rho >0$ and $n \rightarrow \infty$. Note that for $\rho =\frac{\bar{\rho }}{n}$ and $n \rightarrow \infty$, every individual forms new partnerships with individuals already in the population at total rate $\bar{\rho }$. This rate is independent of the number of partners the individual (or his or her new partner) already has. Thus avoiding the source of dependence between individuals in the model with a bounded number of partners per individual.

We assume that an individual enters the population with an expected number $\mu _{\text {in}}$ of partners. and consider the stationary distribution of an individual’s number of partners. Individuals acquire new partners at rate $\bar{\rho } + \mu \mu _{\text {in}}$ (note that this is independent of the number of partners the individual already has) and lose partners at rate $\sigma + \mu$. If the stationary distribution of the number of partners is distributed as *D*, then for $k=0,1, \ldots ,$ the probabilities $d_k = \mathbb {P}(D=k)$ needs to satisfy the following balance equation

10 $$\begin{aligned} (\bar{\rho }+\mu \mu _{\text {in}}) d_k=(\sigma +\mu )d_{k+1}(k+1). \end{aligned}$$

Noting that $\sum ^{\infty }_{k=0}d_{k}=1,$ it follows from (10) that

11 $$\begin{aligned} d_k=\displaystyle \frac{\left( \frac{\bar{\rho }+\mu \mu _{\text {in}}}{\sigma +\mu }\right) ^{k} {\mathrm{e}}^{-\frac{\bar{\rho }+\mu \mu _{\text {in}}}{\sigma +\mu }}}{k!}, \end{aligned}$$

i.e. *D* is Poisson distributed with expectation $\frac{\bar{\rho }+\mu \mu _{\text {in}}}{\sigma +\mu }.$ In order to let the degree of entering individuals be stationary from the start, we want $\mu _\mathrm{in}$ to satisfy the following equation:

$$\begin{aligned} \mu _\mathrm{in}= \mathbb {E}(D) = \frac{\bar{\rho }+\mu \mu _{\text {in}}}{\sigma +\mu }, \end{aligned}$$

which implies $\mu _{\text {in}} = \bar{\rho }/\sigma$. So, *D* is Poisson distributed with expectation $\bar{\rho }/\sigma$. Furthermore, newly arriving individuals also have this degree distribution.

### Threshold parameter

Having determined the degree distribution, we can now compute the expected number of partners infected by one infectious individual. We denote this expected number by $\mathbb {R}_0$, which corresponds to $R_0$ as defined for the finite *n* case.

First we compute the probability that the infectious individual (say $v_1$) infects a given other individual, say $v_2,$ who was already a partner of $v_1$ at the moment $v_1$ got infected. This probability to infect susceptible partner is given by

12 $$\begin{aligned} \int ^{\infty }_{0} \mu {\mathrm{e}}^{-\mu \mathrm{t}}\int ^{\mathrm{t}}_{0} \beta {\mathrm{e}}^{-\beta \mathrm{u}}\mathrm{e}^{-\mu \mathrm{u}}{\mathrm{e}}^{-\sigma \mathrm{u}} \mathop {}\!\mathrm {d}\mathrm{u} \mathop {}\!\mathrm {d}\mathrm{t}=\frac{ \beta }{\beta +\sigma +2\mu }. \end{aligned}$$

Here, 0 can be seen as the time when $v_1$ got infected, *t* is the time when $v_1$ dies and *u* is the time when $v_1$ infects $v_2$. Since the expected number of susceptible partners of an individual at the time of infection is $\bar{\rho }/\sigma$, the expected number of partners $v_1$ infects, among those individuals who were already partners at the time $v_1$ was infected is

13 $$\begin{aligned} \frac{\bar{\rho }}{\sigma } \int ^{\infty }_{0} \mu {\mathrm{e}}^{-\mu {\mathrm{t}}}\int ^{\mathrm{t}}_{0} \beta {\mathrm{e}}^{-\beta \mathrm{u}}\mathrm{e}^{-\mu \mathrm{u}}{\mathrm{e}}^{-\sigma \mathrm{u}}\mathop {}\!\mathrm {d}\mathrm{u} \mathop {}\!\mathrm {d}\mathrm{t} = \frac{\bar{\rho } \beta }{\sigma (\beta +\sigma +2\mu )}. \end{aligned}$$

Similarly, we can compute the probability that an individual $v_1$ who dies at time *t* after being infected, infects a partner $v_2$ which it contacts at time *s* since $v_1$ got infected ($s<t$). This probability is given by

$$\begin{aligned} \int ^{t}_{s} \beta {\mathrm{e}}^{-\beta (\mathrm{u-s})}{\mathrm{e}}^{-\mu (\mathrm{u-s})}{\mathrm{e}}^{-\sigma (\mathrm{u-s})}\mathop {}\!\mathrm {d}\mathrm{u}. \end{aligned}$$

So, using the fact that the total rate an individual acquires new partners is $(\bar{\rho }+\mu \frac{\bar{\rho }}{\sigma })$, the expected number of individuals $v_1$ infects, among those individuals who were not yet partners of $v_1$ at the time $v_1$ got infected is given by

14 $$\begin{aligned} \int ^{\infty }_{0} \mu {\mathrm{e}}^{-\mu \mathrm{t}}\int ^{\mathrm{t}}_{0}\left( \bar{\rho }+\mu \frac{\bar{\rho }}{\sigma }\right) \int ^{\mathrm{t}}_{\mathrm{s}} \beta {\mathrm{e}}^{-\beta ({\mathrm{u-s}})}{\mathrm{e}}^{-\mu ({\mathrm{u-s}})}{\mathrm{e}}^{-\sigma ({\mathrm{u-s}})}\mathop {}\!\mathrm {d}\mathrm{u} \mathop {}\!\mathrm {d}\mathrm{s} \mathop {}\!\mathrm {d}\mathrm{t} = \frac{\bar{\rho }\beta (\sigma +\mu )}{\sigma \mu (\beta +\sigma +2\mu )}. \end{aligned}$$

Combining the above two observations (13) and (14), we arrive at the following expression for the basic reproduction number:

15 $$\begin{aligned} \mathbb {R}_0 = \frac{\bar{\rho } \beta }{\sigma (\beta +\sigma +2\mu )}+ \frac{\bar{\rho }\beta (\sigma +\mu )}{\sigma \mu (\beta +\sigma +2\mu )} = \frac{\bar{\rho }\beta (\sigma + 2\mu )}{\sigma \mu (\beta +\sigma +2\mu )}. \end{aligned}$$

#### Remark

Altmann (1995) considers a model very similar to ours but not exactly the same. He considers an SIR epidemic in a population in which individuals do not die but recover (and acquire eternal immunity) and no new individuals can enter the population. It is easy to check that $\mathbb {R}_0$ in (15) obtained above is in agreement with the result in equation (1) of Altmann (1995) after setting the death rate of partners to 0, which leads to replacing $\beta +\sigma +2\mu$ by $\beta +\sigma +\mu$ in the denominators of both terms in the middle expression of (15) and dropping the factor $(\sigma + \mu )/\sigma$ in the second term of the middle expression of (15).

### Outbreak probability

In order to find the probability of a minor outbreak, we need the distribution of the number of new infectious binding sites that are generated by each infected individual. This then defines the offspring distribution for our branching process.

Assume that individual $v_1$ is infectious for *t* time units. We have already computed the probability of infecting a given other individual who was already a partner of $v_1$ at the moment $v_1$ got infected [see (12)]. Conditioned on *t*, this probability is

$$\begin{aligned} \int ^{{t}}_{0} \beta e^{-\beta u}e^{-\mu u}e^{-\sigma u}\mathop {}\!\mathrm {d}u = \frac{\beta }{\alpha }\left( 1-e^{-\alpha t}\right) , \end{aligned}$$

where $\alpha =\beta +\mu +\sigma$. Furthermore, conditioned on *t*, whether a given partner of $v_1$ at the time $v_1$ got infected (say time 0), will itself be infected by $v_1$ is independent of which other individuals $v_1$ infects. This implies that the probability generating function of $Z_1(t)$, the number of partners of $v_1$ at time 0, who are ultimately infected by $v_1$ for $s \in [0,1]$ is given by

16 $$\begin{aligned} \mathbb {E}\left( s^{Z_1(t)}\right) =\sum _{\ell =0}^{\infty } \frac{\left( \frac{\bar{\rho }}{\sigma }\right) ^{\ell }}{\ell !}e^{-\frac{\bar{\rho }}{\sigma }} \left( \frac{\beta }{\alpha }\left( 1-e^{-\alpha t}\right) s+1-\frac{\beta }{\alpha }\left( 1-e^{-\alpha t}\right) \right) ^{\ell }. \end{aligned}$$

Still assuming that $v_1$ lives until time *t* since infection, $v_1$ can also infect individuals that are not yet partners of $v_1$ at time 0. As described above, an individual acquires new partners according to a homogeneous Poisson process with intensity $\bar{\rho } \frac{\sigma + \mu }{\sigma }$. Up to time *t*, the distribution of the number of acquired partners is therefore Poisson distributed with expectation $\bar{\rho } \frac{\sigma + \mu }{\sigma }$. If we condition on $v_1$ acquiring *m* partners in the time interval (0, *t*), then, by standard properties of the Poisson process (Resnick 2013, Section 4.5), those *m* time points are distributed as *m* independent uniformly distributed random variables on (0, *t*). Let $Z^{\prime }_2(t,m)$ be the random number of individuals $v_1$ infects that were not partners yet at time 0, conditioned on $v_1$ dying at time *t* and acquiring *m* partners in (0, *t*). This argument shows that

17 $$\begin{aligned} \mathbb {E}\left( s^{Z^{\prime }_2(t,m)}\right) = \left( \frac{1}{t}\int _{0}^{t}\left( \frac{\beta }{\alpha } \left( 1-e^{-\alpha (t-u)}\right) s+1-\frac{\beta }{\alpha } \left( 1-e^{-\alpha (t-u)}\right) \right) \mathop {}\!\mathrm {d}u \right) ^{m}. \end{aligned}$$

Further, let $Z_2(t)$ be the random number of individuals $v_1$ infects which were not partners yet at time 0, conditioned on $v_1$ dying at time *t*, not conditioned on the number of partners acquired in (0, *t*). We obtain:

18 $$\begin{aligned} \mathbb {E}(s^{Z_2(t)})= & {} \sum _{m=0}^{\infty } \frac{\left( \frac{\bar{\rho }(\sigma +\mu )t}{\sigma }\right) ^{m}}{m!}e^{-\frac{\bar{\rho }(\sigma +\mu )t}{\sigma } }\mathbb {E}\left( s^{Z^{\prime }_2(t,m)}\right) ,\nonumber \\= & {} \sum _{m=0}^{\infty } \frac{\left( \frac{\bar{\rho }(\sigma +\mu )t}{\sigma }\right) ^{m}}{m!}e^{-\frac{\bar{\rho }(\sigma +\mu )t}{\sigma } }\left( \frac{1}{t}\int _{0}^{t}\left( \frac{\beta }{\alpha } \left( 1-e^{-\alpha (t-u)}\right) s \right. \right. \nonumber \\&\left. \left. +1-\frac{\beta }{\alpha }\left( 1-e^{-\alpha (t-u)}\right) \right) \mathop {}\!\mathrm {d}u \right) ^{m}. \end{aligned}$$

Note that because we assume $n= \infty$, conditioned on *t*, $Z_1(t)$ and $Z_2(t)$ are independent of each other, which implies that $\mathbb {E}(s^{Z_1(t) +Z_2(t)}) = \mathbb {E}(s^{Z_1(t)})\mathbb {E}(s^{Z_2(t)}).$ Let *Z* be the random variable describing the total number of individuals infected by $v_1$. By integrating over time, we obtain by (16) and (18) that:

19 $$\begin{aligned} \mathbb {E}(s^{Z})= & {} \int _{0}^{\infty } \mu e^{-\mu t} \mathbb {E}\left( s^{Z_1(t)}\right) \mathbb {E}\left( s^{Z_2(t)}\right) \mathop {}\!\mathrm {d}t,\nonumber \\= & {} \int _{0}^{\infty } \mu e^{-\mu t}\left( \sum _{l=0}^{\infty } \frac{\left( \frac{\bar{\rho }}{\sigma }\right) ^{\ell }}{\ell !} e^{-\frac{\bar{\rho }}{\sigma }}\left( \frac{\beta }{\alpha }\left( 1-e^{-\alpha t}\right) s+1-\frac{\beta }{\alpha }\left( 1-e^{-\alpha t}\right) \right) ^{\ell }\right) \nonumber \\&\times \left( \sum _{m=0}^{\infty } \frac{\left( \frac{\bar{\rho }(\sigma +\mu )t}{\sigma }\right) ^{m}}{m!}e^{-\frac{\bar{\rho }(\sigma +\mu )t}{\sigma } }\left( \frac{1}{t}\int _{0}^{t} \left( \frac{\beta }{\alpha }\left( 1-e^{-\alpha (t-u)}\right) s \right. \right. \right. \nonumber \\&\quad \left. \left. \left. +1-\frac{\beta }{\alpha }\left( 1-e^{-\alpha (t-u)}\right) \right) \mathop {}\!\mathrm {d}u\right) ^{m}\right) \mathop {}\!\mathrm {d}t. \end{aligned}$$

where $\mathbb {E}(s^{Z})$ is the probability generating function of *Z*. Equation (19) can further be simplified as follows.

20 $$\begin{aligned} \mathbb {E}(s^{Z})= & {} \int _{0}^{\infty } \mu e^{-\mu t}e^{-\frac{\bar{\rho }}{\sigma }}\sum _{\ell =0}^{\infty } \frac{\left( \frac{\bar{\rho }}{\sigma }\right) ^{\ell }}{\ell !} \left( 1-\frac{\beta }{\alpha }\left( 1-e^{-\alpha t}\right) (1-s)\right) ^{\ell }\nonumber \\&\times e^{-\frac{\bar{\rho }(\sigma +\mu )t}{\sigma }}\sum _{m=0}^{\infty } \frac{\left( \frac{\bar{\rho }(\sigma +\mu )}{\sigma } t\right) ^{m}}{m!}\left( 1-\frac{\beta }{\alpha } (1-s)+\frac{\beta }{\alpha ^{2}t}\left( 1-e^{-\alpha t}\right) (1-s)\right) ^{m}\mathop {}\!\mathrm {d}t,\nonumber \\= & {} \displaystyle \int _{0}^{\infty } \mu e^{-\mu t}e^{-\frac{\bar{\rho }\beta }{\sigma \alpha }\left( 1-e^{-\alpha t}\right) (1-s)}e^{-\frac{\bar{\rho }\beta }{\sigma \sigma }(\sigma +\mu )\left( t-\frac{1}{\alpha }\left( 1-e^{-\alpha t}\right) \right) (1-s)}\mathop {}\!\mathrm {d}t. \end{aligned}$$

To simplify notation, we write, recalling that $\alpha =\beta +\mu +\sigma$, that $c=\frac{\bar{\rho }\beta (\sigma +\mu )}{\sigma \alpha }$ and $d=\frac{\bar{\rho }\beta }{\sigma \alpha } (1-\frac{\sigma +\mu }{\alpha })=\frac{\bar{\rho }\beta ^{2}}{\sigma \alpha ^{2}}.$ After rearranging the terms in (20), a little algebra yields:

21 $$\begin{aligned} \mathbb {E}(s^{Z})= & {} \displaystyle \mu e^{-d(1-s)} \int _{0}^{\infty } e^{-\left( \mu +c(1-s)\right) t}e^{d(1-s)e^{-\alpha t}}\mathop {}\!\mathrm {d}t,\nonumber \\= & {} \displaystyle \mu e^{-d(1-s)} \int _{0}^{\infty } \sum _{\ell =0}^{\infty }\frac{\left( d(1-s)\right) ^{\ell }}{\ell !} e^{-\left( \mu +c(1-s)+\ell \alpha \right) t}\mathop {}\!\mathrm {d}t, \nonumber \\= & {} \displaystyle \mu e^{-d(1-s)}\sum _{\ell =0}^{\infty }\frac{\left( d(1-s)\right) ^{\ell }}{\ell !(\mu +c(1-s)+\ell \alpha )}. \end{aligned}$$

Thus, we have found an expression for the probability generating function for the number of offspring generated by an infectious individual, involving an infinite series with infinite radius of convergence. Note that, the probability $\mathbb {P}(Z=k)$ for a specific *k* can be determined from the probability generating function through

$$\begin{aligned} \mathbb {P}(Z=k) = \frac{1}{k!} \frac{d^k}{ds^k} \mathbb {E}(s^{Z})|_{s=0}, \end{aligned}$$

but explicit expressions for these probabilities are long and hardly insightful.

Furthermore, we can also find the probability of extinction of the branching process as the smallest positive root of $s=\mathbb {E}(s^{Z})$. Again, there is no nice closed form expression for this root, however it can be approximated numerically.

### Comparison of $R_0$ and $\mathbb {R}_0$

Finally, we compare $$\mathbb {R}_0$$ in the model with the infinite partnership capacity to the basic reproduction number $$ R_0$$ of Sect. 3.1 in the limit of large partnership capacity. In the limit $$n \rightarrow \infty $$, the asymptotic fraction of free binding sites as described in Eq. (2) becomes

$$\begin{aligned} F= & {} \displaystyle \frac{-\sigma +\sigma \sqrt{1+4\frac{\bar{\rho }}{n\sigma }}}{2\frac{\bar{\rho }}{n}},\\= & {} \frac{n}{2\bar{\rho }}\left( -\sigma +\sigma \left( 1+\frac{2\bar{\rho }}{\sigma n}-\frac{2\bar{\rho }^{2}}{\sigma ^{2} n^{2}}+o(1/n^{2})\right) \right) = 1-\frac{\bar{\rho }}{\sigma n} + o(1/n), \end{aligned}$$

where a function $g(x) =o(x)$ if $g(x)/x \rightarrow 0$ for $x \rightarrow 0$. Therefore, $F \rightarrow 1-\frac{\bar{\rho }}{\sigma n}$ as $n\rightarrow \infty .$ Using $F \rightarrow 1-\frac{\bar{\rho }}{\sigma n}$ in (4), a little algebra confirms that

$$\begin{aligned} \lim _{n\rightarrow \infty }R_0=\displaystyle \frac{\bar{\rho }\beta (\sigma +2\mu )}{\sigma \mu (\beta +\sigma +2\mu )}, \end{aligned}$$

which agrees with Eq. (15).

## Conclusion

The reproduction number and the probability of extinction are among the most fundamental concepts in the theory of mathematical modelling of the spread of infectious diseases. These quantities have importance for health officials for planning and allocation of funds to control the spread of those diseases. We explored different strategies to derive explicit expressions for these two important quantities for an *SI* epidemic on a dynamic sexual network using branching processes. Although it is difficult to derive analytical expressions for threshold conditions and the probability of extinction for a disease spreading on a dynamic network, the branching process approach provide insights for determining the analytical expressions both for the threshold quantity and the probability of extinction. To derive these quantities, we proposed two approaches.

In the first approach, we considered the case in which every individual has *n* binding sites. This approach suffers from some undesired dependencies, as a result we ended up with an approximating branching process that in fact was not an asymptomatically exact approximation of the original epidemic process. The dependencies are demonstrated in detail and an example is provided to clarify the dependencies that violate the (for branching processes) crucial independence criterion of reproducing individuals. The obtained insights are a warning of dependencies which are easily overlooked.

By the simple modelling framework of this first approach, it is only possible to derive the value of the basic reproduction number $R_0.$ However, the probability of extinction of this approximate branching process is also computed to compare it with the true probability of extinction for the special case in which an individual has at most one partner at a time. Interestingly, starting from one infectious individual, the derivation of $R_0$ does not depend on the fundamental independence criteria of the number of children at different binding sites. This suggests that the corresponding explicitly derived value of $R_0$ is exact. However, this does not guarantee the occurrence of a major outbreak with positive probability even if the basic reproduction number $R_0>1.$ This finding is in contrast to classical epidemic models where a major outbreak has strictly positive probability if and only if $R_0>1$.

In the second approach, we demonstrated a simple version of the model in which every individual can have at most one partner at a time. For this model, we managed to establish an asymptotically exact branching process approximation and derived the offspring distribution of this branching process, which allows us to easily compute the probability of extinction for the branching process (and thus for the epidemic). The expectation of the offspring distribution is a threshold parameter. Finally, for $n=1$, it is verified that the epidemic threshold parameters obtained by the two different schemes are the same.

In deriving our models and sticking to branching process approximation as a tool for the analysis, we find that the dependence has a subtle influence on the approximations of the epidemic process by branching processes. This dependence disappears if $n=\infty$. In that case we can compute the basic reproduction number $\mathbb {R}_0$ and the degree distribution of the number of partners of an individual. The probability generating function of the distribution of the number of offspring produced by an infectious individual, that involve a convergent infinite series, is also calculated. This helps us derive an implicit expression of extinction probability. Moreover, we show that our computations are consistent in the sense that for $n \rightarrow \infty$, $R_0 \rightarrow \mathbb {R}_0.$

The current study is only a first step in studying the spread of the disease on a dynamic network using a branching process approach. In future work, we hope to further investigate the disease dynamics by dropping the stationary distribution assumption of the number of partners at debut.

## Appendix A: Convergence of the fraction of free binding sites

In this appendix, we prove that the fraction of free binding sites in a population with size parameter *N*, say $F_N(t)$, indeed (under certain conditions) converges to a constant *F* with probability tending to 1 as $N \rightarrow \infty$. To do this we consider a sequence of the epidemic processes indexed by the expected population size *N*. We often write that an event occurs w.h.p. (with high probability), which means that the probability of the event converges to 1 as $N \rightarrow \infty$ and $t \rightarrow \infty$.

Since, for finite *N* the number of edges in the graph will within finite (but exponentially large) time have been both 0 and *nN*, we cannot obtain that $F_N(t) \rightarrow F$ as $t \rightarrow \infty$ with high probability. So, we settle for proving that for *s* large enough and every $\varepsilon >0$ and $t>0$ we have that $\sup _{s \le u \le s+ t} {\left| F_N(u)-F\right| } < \varepsilon$, with high probability. To do this, we prove that for all $\varepsilon >0$ and all $t>0$, $\sup _{u \le t} {\left| F_N(u)-F(u)\right| }< \varepsilon$ w.h.p., where *F*(*t*) satisfies

22 $$\begin{aligned} F(t) = F(0) - \int _0^t 2 \mu \left( p_{\text {in}}-(1-F(s))\right) - \sigma (1-F(s)) + \rho (F(s))^2 \mathop {}\!\mathrm {d}s \quad \text{ for } t\ge 0. \end{aligned}$$

Here and throughout this appendix we assume that $F_N(0) \rightarrow F(0)$ as $N \rightarrow \infty$. The proof we provide is inspired by the proof of (Ethier and Kurtz 2009, Thm 11.2.1, p. 456). The key distinction between our proof and the proof presented in Ethier and Kurtz (2009) is that $F_N(t)$ is the number of free binding sites divided by the total number of binding sites at time *t*, where the latter is proportional to the population size at time *t* and not to the expected population size *N*. Because of this minor discrepancy, our model cannot exactly be written in terms of equations (2.1)–(2.3) of (Ethier and Kurtz 2009, p. 455) and all steps in the proof have to be checked for our model.

Let $N^*_N(t)$ be the actual population size at time *t* and recall that the stationary distribution of $N^*_N(t)$ is Poisson distributed with expectation *N*. Throughout we assume that

23 $$\begin{aligned} {\left| N^*_N(0)-N\right| }\le N^{2/3}. \end{aligned}$$

From (Ethier and Kurtz 2009, Thm 11.2.3) we then deduce that for all $t>0$,

24 $$\begin{aligned} {\left| N^*_N(s)-N\right| } \le 2N^{2/3} \quad \text{ for } \text{ all } s \in [0,t] \text{ w.h.p. } \end{aligned}$$

Note that (24) implies that,

25 $$\begin{aligned} {\left| \frac{N^*_N(s_1)}{N^*_N(s_2)} -1\right| } \le 5 N^{-1/3} \quad \text{ for } \text{ all } s_1,s_2 \in [0,t] \text{ w.h.p. } \end{aligned}$$

Let $M_N(t)$ be the number of partnerships in the population at time *t*. Because each partnership involves two individuals, the sum of the number of partners over all individuals is therefore $2M_N(t)$. Furthermore,

$$\begin{aligned} F_N(t)= 1-\frac{2 M_N(t)}{nN^*_N(t)}. \end{aligned}$$

There are four events which change the number of partnerships in the population.

(i) A new individual entering the population, which leads to an expected increase of $n p_{\text {in}}$ to the number of partnerships and occurs at intensity $\mu N$.
(ii) An individual dies, which leads to an expected decrease of $\frac{2 M_N(t)}{N^*_N(t)}$ to the number of partnerships and occurs at (time dependent) intensity $\mu N_N^*(t)$.
(iii) Separation, which decreases the number of partnerships by 1 and occurs at intensity $\sigma M_N(t)$ and
(iv) Formation of a new pair, which increases the number of partnerships by 1 and occurs at intensity
  $$\begin{aligned} \frac{\rho }{2} (nN^*_N(t)-2M_N(t)) \frac{nN^*_N(t)-2M_N(t)}{nN^*_N(t)} = \frac{\rho n N^*_N(t)}{2} \left( 1-\frac{2 M_N(t)}{nN^*_N(t)}\right) ^2. \end{aligned}$$

Denote the times at which one of the above events occur by $t_1< t_2< \cdots$ and set $t_0=0$. Let

$$\begin{aligned} \iota (t) =\max \left\{ i \ge 0;t_i \le t\right\} , \end{aligned}$$

be the number of events up to time *t*. Define

26 $$\begin{aligned} \lambda (t) = \frac{nN^*_N(t)}{2} \left( 2 \mu \frac{N}{n N^*_N(t)} + \frac{2 \mu }{n} + \sigma \frac{2 M_N(t)}{n N^*_N(t)} + \rho \left( 1-\frac{2 M_N(t)}{nN^*_N(t)}\right) ^2\right) . \end{aligned}$$

That is, $\lambda (t)$ is the rate at which the first event after time *t* occurs. By (24) we have

27 $$\begin{aligned} 0< \frac{2 \mu }{n} \le \frac{2}{nN^*_N(s)}\lambda (s) \le \frac{5 \mu }{n} + \sigma + \rho < \infty \quad \text{ for } \text{ all } s \in [0,t] \text{ w.h.p. } \end{aligned}$$

Let $\mathscr {F}_i$ be the $\sigma$-algebra generated by the whole dynamic random graph process up to time $t_i$. So $\{(M_N(t),N^*_N(t))\}_{t \in [0,t_i]}$ is measurable with respect to $\mathscr {F}_i$. For $i \ge 0$, the interarrival time $t_{i+1}-t_i$ is exponentially distributed with parameter $\lambda (t_i)$. Conditioned on $\{\lambda (t_i)\}_{i \ge 0}$, the interarrival times are independent. For $i\ge 1$, let $J_i= M_N(t_{i})-M_N(t_{i-1})$ and note that $J_i \in [-n,n]$ (i.e. the maximal instantaneous change in $M_N(t)$ is *n*) and that

$$\begin{aligned} \hat{J}_i= & {} \mathbb {E}\left[ J_i|\mathscr {F}_{i-1}\right] = \frac{2 \mu \frac{N}{N^*_N(t_{i-1})}p_{\text {in}} - 2 \mu \frac{2 M_N(t_{i-1})}{n N^*_N(t_{i-1})} - \sigma \frac{2 M_N(t_{i-1})}{n N^*_N(t_{i-1})} + \rho \left( 1-\frac{2 M_N(t_{i-1})}{nN^*_N(t_{i-1})}\right) ^2}{2 \mu \frac{N}{n N^*_N(t_{i-1})} + \frac{2 \mu }{n} + \sigma \frac{2 M_N(t_{i-1})}{n N^*_N(t_{i-1})} + \rho \left( 1-\frac{2 M_N(t_{i-1})}{nN^*_N(t_{i-1})}\right) ^2}\\= & {} \frac{\mu n N p_{\text {in}} - 2 \mu M_N(t_{i-1}) - \sigma M_N(t_{i-1}) + \rho \frac{nN^*_N(t)}{2} \left( 1-\frac{2 M_N(t_{i-1})}{nN^*_N(t_{i-1})}\right) ^2}{\lambda (t_{i-1})}. \end{aligned}$$

Define

$$\begin{aligned} \hat{M}_i = J_i- (t_i -t_{i-1})\lambda (t_{i-1})\hat{J}_i, \quad \text{ for } i \ge 1 \end{aligned}$$

and note that $\mathbb {E}[\hat{M}_{i+1}|\mathscr {F}_{i}] =0$ and

28 $$\begin{aligned} \mathbb {E}\left[ (\hat{M}_{i+1})^2|\mathscr {F}_i\right] = \mathbb {E}\left[ (J_{i+1})^2|\mathscr {F}_{i}\right] \le 4 n^2. \end{aligned}$$

Because $J_i = 1$ with probability at least

$$\begin{aligned} \frac{\mu N}{\lambda (t_i)} \ge \frac{2 \mu N}{nN^*_N(t)} \frac{1}{\frac{5 \mu }{n} + \sigma + \rho } \ge \frac{2 \mu N}{N+ 2N^{2/3}} \frac{1}{5 \mu + \sigma n + \rho n} \ge \frac{\mu }{5 \mu + \sigma n + \rho n}, \end{aligned}$$

we obtain that with high probability [here we used (27) and (24)],

29 $$\begin{aligned} 0< \frac{\mu }{5 \mu + \sigma n + \rho n} \le \mathbb {E}[(\hat{M}_{i+1})^2|\mathscr {F}_i] \le 4n^2 < \infty . \end{aligned}$$

Furthermore,

$$\begin{aligned} M_N(t)= & {} M_N(0) + \sum _{i=1}^{\iota (t)} \hat{M}_i + \int _0^{\iota (t)} \lambda (s)\hat{J}_{\iota (s)} \mathop {}\!\mathrm {d}s\\= & {} M_N(0) + \sum _{i=1}^{\iota (t)} \hat{M}_i + \int _0^{\iota (t)} \mu nNp_{\text {in}} - (2 \mu + \sigma ) M_N(s) \\&\quad + \rho \frac{nN^*_N(s)}{2} \left( 1-\frac{2 M_N(s)}{nN^*_N(s)}\right) ^2 \mathop {}\!\mathrm {d}s{.} \end{aligned}$$

Writing $F_N(t) = 1-\frac{2 M_N(t)}{n N^*_N(t)}$, this reads

$$\begin{aligned} F_N(t)= & {} F_N(0) \frac{N^*_N(0)}{N^*_N(t)} - \frac{2 \iota (t)}{nN^*_N(t)} \frac{1}{\iota (t)} \sum _{i=1}^{\iota (t)} \hat{M}_i\\&- \int _0^{\iota (t)} 2\mu \frac{N}{N^*_N(t)} p_{\text {in}} - (2\mu + \sigma ) \frac{N^*_N(s)}{N^*_N(t)} (1-F_N(s)) + \rho \frac{N^*_N(s)}{N^*_N(t)} \left( F_N(s)\right) ^2 \mathop {}\!\mathrm {d}s. \end{aligned}$$

So using (22), we obtain

$$\begin{aligned}&{\left| F_N(t)-F(t)\right| } \le {\left| F_N(0) \frac{N^*_N(0)}{N^*_N(t)}-F(0)\right| } +{\left| \frac{\iota (t)}{nN^*_N(t)} \frac{1}{\iota (t)} \sum _{i=1}^{\iota (t)} \hat{M}_i\right| }\\&\quad + {\biggl | \int _0^{\iota (t)} \left[ 2\mu \frac{N}{N^*_N(t)} p_{\text {in}} - (2\mu + \sigma ) \frac{N^*_N(s)}{N^*_N(t)} (1-F_N(s)) + \rho \frac{N^*_N(s)}{N^*_N(t)} \left( F_N(s)\right) ^2\right] } \mathop {}\!\mathrm {d}s\\&\quad -\int _0^t (2 \mu p_{\text {in}} - (2\mu + \sigma )(1-F(s) + \rho (F(s))^2 \mathop {}\!\mathrm {d}s{\biggl |}, \end{aligned}$$

which in turn provides us with the following upper bound

$$\begin{aligned} {\sup _{0 \le u \le t} \left| F_N(u)-F(u)\right| }\le & {} \sup _{0 \le u \le t} {\left| F_N(0) \frac{N^*_N(0)}{N^*_N(u)}-F(0)\right| } \\&+ \sup _{0 \le u \le t} {\left| \frac{N}{N^*_N(u)} \frac{1}{n N} \sum _{i=1}^{\iota (u)} \hat{M}_i\right| }\\&+ \sup _{0 \le u \le t} {\left| \int _{t_{\iota (u)}}^u (2 \mu p_{\text {in}} - (2\mu + \sigma )(1-F(s) + \rho (F(s))^2 \mathop {}\!\mathrm {d}s\right| }\\&+ \sup _{0 \le u \le t} \int _0^{t_{\iota (u)}} 2 \mu p_{\text {in}} {\left| \frac{N}{N^*_N(u)}-1\right| }\mathop {}\!\mathrm {d}s \\&+ \sup _{0 \le u \le t} \int _0^{t_{\iota (u)}} (2\mu + \sigma ){\left| \frac{N^*_N(s)}{N^*_N(u)}(1-F_N(s))-(1-F(s))\right| }\mathop {}\!\mathrm {d}s\\&+ \sup _{0 \le u \le t} \int _0^{t_{\iota (u)}} \rho {\left| \frac{N^*_N(s)}{N^*_N(u)} \left( F_N(s)\right) ^2 -(F(s))^2\right| } \mathop {}\!\mathrm {d}s. \end{aligned}$$

We can now analyse the six terms on the right hand side separately.

1. We may bound the first term as follows,
  $$\begin{aligned} {\left| F_N(0) \frac{N^*_N(0)}{N^*_N(u)}-F(0)\right| } \le F_N(0){\left| \frac{N^*_N(0)}{N^*_N(u)}-1\right| } + {\left| F_N(0)-F(0)\right| }. \end{aligned}$$
  For all $0<u \le t$ the first term on the right hand side is w.h.p. bounded by $5N^{-1/3}$ [using (25)]. The second term converges to 0 because $F_N(0) \rightarrow F(0)$ as $N \rightarrow \infty$.
2. For $\sup _{0 \le u \le t} |\frac{N}{N^*_N(u)} \frac{1}{n N} \sum _{i=1}^{\iota (u)} \hat{M}_i|$, note that $\sup _{0 \le u \le t} |\frac{N}{N^*_N(u)}| < 2$ w.h.p. by (24).
  By Kolmogorov’s inequality [see (Durrett 2010, Thm 2.5.2)] we obtain that
  $$\begin{aligned} \mathbb {P}\left( \max _{i\le k \le \iota (u)} {\left| \sum _{i=1}^{k} \hat{M}_i\right| } \ge n N^{2/3} \right) \le N^{-4/3} \iota (u). \end{aligned}$$
  So, for $$u \le t$$
  $$\begin{aligned} \mathbb {P}\left( \max _{i\le k \le \iota (u)} \frac{1}{nN}{\left| \sum _{i=1}^{k} \hat{M}_i\right| } \ge N^{-1/3} \right) \le N^{-4/3} \iota (u) \le N^{-4/3} \iota (t). \end{aligned}$$
  Furthermore, $\iota (t)$ is the number of events up to time *t*, which, by $N^*_N(u) < 2N$ for all $u \in [0,t]$ w.h.p. and by (24) and (27), is w.h.p. bounded above by a Poisson distributed random variable with expectation $n N t\left( \frac{5 \mu }{n} + \sigma + \rho \right)$. This bound is distributed as the sum of *N* i.i.d. Poisson distributed random variables with mean $n t\left( \frac{5 \mu }{n} + \sigma + \rho \right)$. So, by the (weak) law of large numbers
  30 $$\begin{aligned} \frac{\iota (t)}{N} \le nt\left( \frac{5 \mu }{n} + \sigma + \rho \right) +1\quad \text{ w.h.p. } \end{aligned}$$
  Combining the above, we obtain that there exists a positive constant $\hat{C}$ such that
  $$\begin{aligned} \sup _{0 \le u \le t} {\left| \frac{N}{N^*_N(u)} \frac{1}{n N} \sum _{i=1}^{\iota (u)} \hat{M}_i\right| } \le \hat{C} n^{-1/3} \quad \text{ w.h.p. } \end{aligned}$$
3. Now observe,
  $$\begin{aligned}&{\left| \int _{t_{\iota (u)}}^u (2 \mu \left( p_{\text {in}}-(1-F(s))\right) - \sigma (1-F(s) + \rho (F(s))^2 \mathop {}\!\mathrm {d}s\right| }\\&\quad \le (u-t_{\iota (u)}) \sup _{t_{\iota (u)} \le s \le u} {\left| (2 \mu \left( p_{\text {in}}-(1-F(s))\right) - \sigma (1-F(s) + \rho (F(s))^2\right| }\\&\quad \le (u-t_{\iota (u)}) (4 \mu + \sigma + \rho ). \end{aligned}$$
  We are interested in
  $$\begin{aligned}&\sup _{0 \le u \le t}{\left| \int _{t_{\iota (u)}}^u (2 \mu \left( p_{\text {in}}-(1-F(s))\right) - \sigma (1-F(s) + \rho (F(s))^2 \mathop {}\!\mathrm {d}s\right| }\\&\quad \le \sup _{0 \le u \le t} (u-t_{\iota (u)}) (4 \mu + \sigma + \rho ). \end{aligned}$$
  Because $u-t_{\iota (u)}$ is exponentially distributed with parameter at least $\mu N/2$ for all *u* in the interval [0, *t*] by (29), $\sup _{0 \le u \le t} (u-t_{\iota (u)})$ is stochastically bounded above by the maximum of $\iota (t)$ independent exponentially distributed random variables with mean at least $\mu N/2$. Denote this maximum by *X*. Let *c*(*N*) be a function depending on *N*, then
  $$\begin{aligned} \mathbb {P}(X > c(N)) = 1 - \left( 1- \frac{1}{e^{\mu N c(N)/2}} \right) ^{\iota (t)}, \end{aligned}$$
  which converges to 0 as $e^{\mu N c(N)/2}/\iota (t) \rightarrow \infty$, which by (30) holds w.h.p. for $c(n) = N^{-1/2}$.
4. By (25) and by $t_{\iota (u)}\le t$ for $u \in [0,t]$ we have
  $$\begin{aligned} \sup _{0 \le u \le t} \int _0^{t_{\iota (u)}} 2 \mu p_{\text {in}} {\left| \frac{N}{N^*_N(u)}-1\right| } \mathop {}\!\mathrm {d}s \le 2 \mu p_{\text {in}} t \left( 5 N^{-1/3}\right) \quad \text{ w.h.p. } \end{aligned}$$
5. Again by (25) and by $t_{\iota (u)} \le u\le t$ for $u \in [0,t]$ we obtain
  $$\begin{aligned}&\sup _{0 \le u \le t} \int _0^{t_{\iota (u)}} (2 \mu + \sigma ) {\left| \frac{N^*_N(s)}{N^*_N(u)}(1-F_N(s))-(1-F(s))\right| } \mathop {}\!\mathrm {d}s\\&\quad \le (2 \mu + \sigma ) \sup _{0 \le u \le t} \int _0^{t_{\iota (u)}} {\left| \frac{N^*_N(s)}{N^*_N(u)}-1\right| }(1-F_N(s)) + {\left| F(s)-F_N(s))\right| } \mathop {}\!\mathrm {d}s\\&\quad \le (2 \mu + \sigma ) t 5N^{-1/3} + (2 \mu + \sigma ) \sup _{0 \le u \le t} \int _0^{u} {\left| F(s)-F_N(s))\right| }\mathop {}\!\mathrm {d}s \quad \text{ w.h.p. } \end{aligned}$$
6. Using (25) and that $t_{\iota (u)} \le u\le t$ for $u \in [0,t]$ for a third time, we see that
  $$\begin{aligned}&\sup _{0 \le u \le t} \int _0^{t_{\iota (u)}} \rho {\left| \frac{N^*_N(s)}{N^*_N(u)} \left( F_N(s)\right) ^2 -(F(s))^2\right| } \mathop {}\!\mathrm {d}s\\&\quad \le \rho \sup _{0 \le u \le t} \int _0^u \left| \left( \frac{N^*_N(s)}{N^*_N(u)}-1\right) \right| \left( F_N(s)\right) ^2 +\left| (F_N(s))^2 -(F(s))^2\right| \mathop {}\!\mathrm {d}s\\&\quad \le \rho \sup _{0 \le u \le t} \int _0^t \left| \left( \frac{N^*_N(s)}{N^*_N(u)}-1\right) \right| ds +\rho \sup _{0 \le u \le t}\int _0^u \left| F_N(s) -F(s)\right| \left| F_N(s) +F(s)\right| \mathop {}\!\mathrm {d}s \\&\quad \le \rho 5 t N^{-1/3} +2 \rho \sup _{0 \le u \le t} \int _0^u \left| F_N(s) -F(s)\right| \mathop {}\!\mathrm {d}s, \quad \text{ w.h.p. } \end{aligned}$$

Combining the above inequalities we obtain that

$$\begin{aligned} \sup _{0 \le u \le t} \left| F_N(u)-F(u)\right| \le \varepsilon (N) + (2 \mu + \sigma +2 \rho ) \sup _{0 \le u \le t} \int _0^{u} \left| F_N(s))-F(s)\right| \mathop {}\!\mathrm {d}s, \quad \text{ w.h.p. } \end{aligned}$$

where $\varepsilon (N) \rightarrow 0$ as $N \rightarrow \infty$. It follows now by Gronwall’s inequality [see (Ethier and Kurtz 2009, Appendix)] that for all $t>0$,

$$\begin{aligned} \sup _{0 \le u \le t} |F_N(u)-F(u)| \rightarrow 0 \end{aligned}$$

in probability as $N \rightarrow \infty$. In particular, there exists $s_0$ such that for all $\varepsilon >0$, all $t>0$ and all $s> s_0$

$$\begin{aligned} \sup _{s \le u \le s+t} \left| F_N(u)-F\right| < \delta , \quad \text{ w.h.p. } \end{aligned}$$

Here we have used that $F(t)\rightarrow F$ as $t \rightarrow \infty$.

## Appendix B: Branching process approximation ignoring dependencies

In this appendix we derive some of the results for the model presented in Sect. 3.1. Recall that in that subsection we approximate the epidemic by a branching process in which we ignore dependencies between the offspring sizes of different individuals. We do incorporate the dependence between binding sites of the same individual, which occurs through death of the individual, but that is the only dependence which appears in the branching process. We refer back to Fig. 1 for a flow chart of the Markov process governing the dynamics of the states of a binding site.

Recall that

$\pi _{\phi }$: Probability that a $\phi$ binding site becomes $+$ before it disappears, i.e. before the individual under consideration dies.
$\pi _-$: Probability that a − binding site becomes $+$ before it disappears.
$\pi _+$: Probability that a $+$ binding site becomes $+$ again after having been − or $\phi$ before it disappears.

Using the transition probabilities represented in Fig. 1, we obtain the following set of equations.

31 $$\begin{aligned} \begin{aligned} \pi _\phi&=\frac{\rho F+\frac{\mu (1-F)}{F}}{\mu +\rho F+\frac{\mu (1-F)}{F}}\pi _-,\\ \pi _-&=\frac{\beta }{\beta +\sigma +2\mu }+\frac{\sigma +\mu }{\beta +\sigma +2\mu }\pi _\phi ,\\ \pi _+&=\frac{\sigma +\mu }{\sigma +2\mu }\pi _\phi .\\ \end{aligned} \end{aligned}$$

Recalling equation (1), we deduce from system (31) that

32 $$\begin{aligned} \pi _\phi =\frac{\beta (\sigma +\mu ) (1-F)}{D}, \quad \pi _- = \frac{\beta (\sigma (1-F)+\mu )}{D} \quad \text{ and } \quad \pi _+ = \frac{\beta (\sigma +\mu )^{2} (1-F)}{(\sigma +2\mu )D}, \end{aligned}$$

where

$$\begin{aligned} D=\beta \left( \sigma (1- F)+\mu )+\mu (\sigma +\mu +\mu F\right) . \end{aligned}$$

In order to derive the offspring distribution of a newly infected individual, we let $X_{\phi }$, $X_{-}$ and $X_{+}$ be the random variables denoting, respectively, the number of infectious binding sites generated by an infected individual who starts in $\phi$, − and $+$ state. We derive the following probability distributions for $\ell =0,1,2,\ldots$

33 $$\begin{aligned} \begin{aligned} \mathbb {P}(X_{\phi }=1+\ell )&=\pi _\phi \pi ^{\ell }_{+}(1-\pi _+),\\ \mathbb {P}(X_{-}=1+\ell )&=\pi _-\pi ^{\ell }_{+}(1-\pi _+),\\ \mathbb {P} (X_{+}=\ell )&=\pi ^{\ell }_{+}(1-\pi _+)\\ \end{aligned} \end{aligned}$$

and $$\mathbb {P}(X_{\phi }=0)=1-\pi _{\phi }$$ and $$\mathbb {P}(X_{-}=0)=1-\pi _-$$.

Thus, if $n=1$, when every individual is “born” in state $+$ (because at the time of infection the individual’s binding site is occupied by his or her infector), the offspring distribution in our process is geometric with parameter $1-\pi _+.$

We immediately obtain from (33) that

34 $$\begin{aligned} \mathbb {E}(X_{\phi })=\frac{\pi _{_\phi }}{1-\pi _+}, \quad \mathbb {E}(X_{-})=\frac{\pi _{-}}{1-\pi _+} \quad \text{ and } \quad \mathbb {E}(X_{+})=\frac{\pi _{+}}{1-\pi _+}. \end{aligned}$$

Let $R_0$ be the expected number of new infectious binding sites generated by one infectious individual in the early stages of an epidemic. A newly infected individual in the early stages of an outbreak starts his or her infectious period with one infected (the infector) and a random number $K_{s}$ of susceptible binding sites. Note that, by the dynamic network properties and because we consider the early stages of an outbreak, $K_s$ is binomially distributed with parameters $n-1$ and $1-F$. Therefore, by this interpretation, $R_0$ is given by

35 $$\begin{aligned} R_0&=\mathbb {E}(\text {number of infections caused by one infectious individual})\nonumber \\&=\mathbb {E}\left( \mathbb {E}(\text {number of infections caused by one infectious individual}\mid K_{s})\right) \nonumber \\&=\mathbb {E}\left( \mathbb {E}(X_+)+ K_{s}\mathbb {E}(X_-)+(n-K_{s}-1)\mathbb {E}(X_{\phi })\right) \nonumber \\&=\mathbb {E}\left( \frac{\pi _+}{1-\pi _+}+K_{s} \frac{\pi _-}{1-\pi _+}+(n-K_{s}-1)\frac{\pi _\phi }{1-\pi _+}\right) \nonumber \\&=\displaystyle \frac{\pi _+}{1-\pi _+}+\frac{n-1}{1-\pi _+} \left( \pi _-(1-F)+\pi _{\phi }F\right) \nonumber \\&=\displaystyle \frac{\beta (1-F)}{\mu (\sigma +\mu +\mu F)(\beta +\sigma +2\mu )}\left( (\sigma +\mu )^{2}+ (n-1)(\sigma +2\mu )(\sigma +\mu +\mu F)\right) . \end{aligned}$$

As stated in Sect. 3.1 the branching process approximation of the epidemic presented in this appendix is not asymptotically exact. In order to to show that ignoring dependencies in the epidemic process really has a substantial effect, we compare in Sect. 3.2 the probability of extinction of this branching process with the correct probability of a minor outbreak of the epidemic as $N \rightarrow \infty$. In order to compute this correct probability of a minor outbreak using the methods of Sect. 3.2, we have to assume that $n=1$. What is left to do is to compute the probability of extinction of the branching process described in Sect. 3.1 for $n=1$.

Standard results from the theory on branching processes (Jagers 1975) give that the extinction probability *q* of a branching process originating from one case, with his or her offspring distributed as $X_+$, is the smallest non-negative fixed point of the offspring generating function $G(s)=\sum ^{\infty }_{i=0}p_{i}s^{i}$ where $p_{i}=P(X_{+}=i)$ and $0 \le s \le 1.$

Recall that at the moment an individual gets infected, he or she has 1 infectious partner (namely his or her parent). From (33), we know that an infected individual has $\ell$ ($\ell =0,1,2,\ldots$) children with probability $\mathbb {P}(X_{+}=\ell ) = \pi ^{\ell }_{+}(1-\pi _+)$. So, the extinction probability, denoted here by $q_{+},$ is given by the smallest root of

$$\begin{aligned} q_+ = \sum ^{\infty }_{\ell =0} \pi ^{\ell }_{+}(1-\pi _+) (q_+)^{\ell } = \frac{1-\pi _+}{1-\pi _+q_+}. \end{aligned}$$

We find that $q_+= \min (1,\frac{1-\pi _+}{\pi _+})$.

In the following we write the extinction probability in terms of $R_0.$ For this, recall from (9) that for $n=1$,

$$\begin{aligned} \beta = \frac{\mu (\sigma +2\mu )(\sigma +\mu +\mu F)R_0}{(\sigma +\mu )^2(1-F)-\mu (\sigma +\mu +\mu F)R_0}. \end{aligned}$$

Using this value of $\beta$ in Eq. (32), we can write $\pi _{\phi }$ and $\pi _{+}$ in terms of $R_0$ as follows:

36 $$\begin{aligned} \pi _{\phi }=\displaystyle \frac{(\sigma +2\mu )R_0}{(\sigma +\mu )(R_0+1)} \quad \text{ and } \quad \pi _{+}=\displaystyle \frac{R_0}{R_0+1}, \end{aligned}$$

where the first equation of (36) implies that we should take $\sigma \ge \mu (R_0-1)$ in order for $\pi _{\phi } \le 1.$ So, if $R_0=\frac{\pi _+}{1-\pi _+}>1,$ then $q_+ =1/R_0$.

If we assume that the branching process starts with an individual with an empty binding site, we can still compute the probability of extinction of the branching process by making use of the following observation. If the initial individual has *k* children, then the offspring of this initial individual only goes extinct if the offspring of the *k* children goes extinct. Those *k* children all correspond to infectious individuals with an infectious binding site at the moment of infection. Recall that

$$\begin{aligned} \mathbb {P}(X_{\phi }=k)= {\left\{ \begin{array}{ll} 1-\pi _{\phi } &{} \text {if }k=0, \\ \pi _{\phi }(1-\pi _{+})\pi _{+}^{k-1} &{} \text {if } k\ge 1. \end{array}\right. } \end{aligned}$$

So, if we denote the probability that the offspring of an individual with an empty binding site goes extinct by $q_{\phi }$, then $q_{\phi }$ satisfies

$$\begin{aligned} q_{\phi } = \sum ^{\infty }_{k=0} \mathbb {P}(X_{\phi }=k) q_+^k = 1-\pi _{\phi } + \frac{\pi _{\phi }(1-\pi _{+})q_+}{1-\pi _{+}q_+}= 1-\pi _{\phi } +\pi _{\phi }q_+^2=\frac{1}{R_0}-\frac{\mu (R_0-1)}{(\sigma +\mu )R_0}. \end{aligned}$$

where we have used (36) and $q_+=1/R_0$ in the last equality. It is not hard to see that if $R_0$ is greater than 1, then the extinction probabilities calculated above are less than 1, which is a minimal requirement for consistency.
